# Body Mass Index or Serum Albumin Levels: Which is further Prognostic following Cardiac Surgery?

**DOI:** 10.5681/jcvtr.2014.026

**Published:** 2014-06-30

**Authors:** Hossein Montazerghaem, Naser Safaie, Vahid Samiei Nezhad

**Affiliations:** ^1^Cardiovascular Research Center, Hormozgan University of Medical Sciences, Bandar Abbas, Iran; ^2^Cardiovascular Research Center, Tabriz University of Medical Sciences, Tabriz, Iran

**Keywords:** Body Mass Index, Serum Albumin, Coronary Artery Bypass Graft Prognosis

## Abstract

***Introduction:*** Patients with low serum albumin and abnormal BMI may be at the risk of death and other complications after surgery. This could be remarkable in patients with coronary arteries bypass graft surgery. Therefore, we decided to evaluate the impact of these factors associated with survival and outcome after cardiac surgery.

***Methods:*** A cross-sectional study was performed from 2009 until 2012 on 345 patients who underwent coronary artery bypass grafts. Also Patients were monitored for a year. Patients’ information was collected and then the patients were analyzed for body mass index (BMI) and serum albumin and their effects on postoperative outcomes. P value <0.05 was considered statistically significant.

***Results:*** Mortality after CABG operation was not of a significant relation in patients with low BMI (BMI <20), normal and high (BMI> 30). Obese patients are more susceptible to myocardial infarction in postoperative period (P=0.02). Pneumonia after surgery in these patients was more common than others (P= 0.023); however, low serum albumin was significantly associated with mortality following operation (P<0.001). Reoperation due to bleeding (P<0.001) and required mechanical ventilation for more than a day (P=0.019) were significantly associated with low serum albumin.

***Conclusion:*** In conclusion, the high or low BMI alone did not increase mortality after cardiac surgery. However, postoperative morbidity in obese patients may be greater than others. Low serum albumin may increase the risk of mortality and postoperative complications as well. Therefore, it seems ameliorating serum albumin can be effective more than body mass index in improving the outcome of patients after CABG surgery.

## Introduction

Coronary artery disease is the most common cardiac pathology which cardiovascular surgeons deal with. The etiology of coronary artery disease is atherosclerosis basically.^1^ It is a multifactorial disease and its main risk factors are hyperlipidemia, smoking, diabetes, hypertension, obesity, sedentary lifestyle and male gender.^1^ Indications for CABG in patients include chronic angina, unstable angina or angina after myocardial infarction in patients with atypical symptoms that ischemia was stimulated during stress test.^2^ Myocardial infarction (MI), bleeding, dysrhythmias, cardiac tamponade, infection, aortic dissection, pneumonia, respiratory failure, renal failure, gastrointestinal complications and multiple organ failure are consequences of surgery.^2^ Exercise capacity after CABG usually improves considerably; therefore, most of patients have better functional response to exercise as long as blood flow improves.^3^ Enhanced performance lasts for 10 years and even for longer in patients who have received Internal Mammary Artery grafts. Long-term survival after CABG is excellent: 5-year survival of 90% and 10-year survival of 75 to 90% depending on comorbidities.^3^ Intensive medical therapy to control diabetes, hypercholesterolemia, and hypertension and smoking significantly improve long-term survival. According to data in STS database (Society of Thoracic Surgeons), the risk of mortality associated with CABG surgery reaches to 1.9% and rises to 15% with severe complications.^3^

Risk of bleeding requiring reoperation is 1.8%, MI 0.9% and the probability of any neurological complications (such as coma, stroke, paralysis, transient ischemic attack) 1.6%. Also, there is 1.1% risk for permanent stroke.^1-3^

Engeiman et al. reported both low body mass index (BMI) and hypoalbuminemia as reason of increased mortality and postoperative complications after cardiac surgery. Thinnest patients had the highest risk factors for postoperative complications.^4^ In a series of studies, BMI was demonstrated to be of a significant effect on the duration of hospitalization of the patient in the ICU and the surgery department. Patients with weight gain and obesity had shorter duration of stay in the ICU than patients with normal BMI and obesity did not have any effects on increase of morbidity and other side effects during operation compared with normal weight.^5,6^

It has been suggested that a lot of attention should be paid to the heart surgery of patients with low BMI due to their being prone to the consequence after the surgery.^7-9^ Habib et al concluded that high rate of deviation in BMI compared to the normal BMI cause direct increase in morbidity and also decrease in long term survival.^10^ Severe obesity (BMI> 40) is specifically related to undesirable consequences escalating duration of hospitalization after CABG.^11-14^ On the other hand, there is belief that obese patients have better consequences and higher survival in hospital than patients with normal BMI.^15,16^

Kesek et al. concluded that cardiac surgery patients who have low BMI have raised mortality rates and low serum albumin levels expand the risk of infection after surgery.^17^

## Materials and methods

A cross-sectional and descriptive-analytic study was conducted in Jorjani heart surgery center of Shahid Mohammadi hospital on patients with CABG by using left internal mammary artery (LIMA) and saphenous vein graft with Cardiopulmonary Bypass (CPB) from June 2009 to August 2012 for 38 months. First of all, patients’ demographic data including patient age, sex, place of birth, place of residence, weight and height as well as laboratory data on admission and risk factors that might affect outcome of the disease were collected. Patients were monitored to evaluate the physical condition of patients and postoperative complications for one year. Some of patients who could not be accessed were excluded from the study.

Patients were sorted in three categories by BMI (low 20> kg/m^2^, normal 20-30 kg/m^2^ and high 30< kg/m^2^). Serum albumin levels were divided into three groups (very low 2.5> g/dl, low 2.5-3.5 g/dl and normal 3.5<g/dl). Data were analyzed using SPSS. 20 and Chi-square test to examine the relationships between variables. P value less than < 0.05 was considered statistically significant.

## Results

Overall, 345 patients with coronary artery bypass graft (CABG) were studied; 59% (203 subjects) were male and 41% (142 subjects) were female. Average age of the patients was 60.7 years; the minimum age was 30 years and maximum 86 years. Of the studied patients, 16.4% aged less than 50 years, 73.4% between 50 and 75 years and 10.2% were over 75 years of age (with the mean weight of 61 kg ranging from 34 to 110 kg). BMI of the patients was between minimum of 13.48 kg/m^2^and maximum 43 kg/m^2^with 23 kg/m^2^ average (21.5% had BMI <20 kg/m^2^, 52.8% with BMI 20 to 25 kg/m^2^, 20% BMI between 25 to 30 kg/m^2^and 5.7% BMI> 30 kg/m^2^).

The mean serum albumin levels was 3.72 g/dl; 0.4% of patients had levels of less than 2.5 g/dl, 36.2% of patients between 2.5 and 3.5 g/dl and 43.4% of patients greater than 3.5 g/dl serum albumin. In terms of gender, 31.9% of females and 39.4% males had low serum albumin levels (2.5-3.5 g/dl). There was no significant relation between gender and serum albumin levels (Table 1).

**Table 1:** Percentage of patients in terms of demographic parameters and blood types and their relation with BMI, serum albumin and mortality

	**Albumin (g/dl)**				**BMI (Kg/m** ^2^ **)**				**Postoperative death**		
	**<2.5**	**2.5-3.5**	**>3.5**	***P***	**<20**	**20-30**	**>30**	***P***	**Yes**	**No**	***P***
**Demographic data**											
**Gender **				0.3				0.04			0.83
Female (%)	0.0	31.9	68.1		15.2	76.1	8.7		6.3	93.7	
Male (%)	0.6	39.4	60		22.7	73.7	3.5		5.7	94.3	
**Percent Age (y)**				0.01				0.006			0.37
<50	0.0	9.4	90.6		2.6	84.2	13.2		4	96	
50-75	0.5	38.6	60.9		20.7	73.9	5.4		5	95	
>75	0.0	50	50		31.4	68.6	0.0		11.5	88.5	
**BMI (Kg/m** ^2^ **)**				0.21							0.20
Percent thin (<20)	0.0	51	49	‏-	‏-	‏-	‏-	‏-	8.3	91.7	‏-
Percent 20-30	0.5	33.7	65.8	‏-	‏-	‏-	‏-	‏-	4.9	95.1	‏-
Percent obese (>30)	0.0	28.6	71.4	‏-	‏-	‏-	‏-	‏-	16.7	83.3	‏-
**Serum albumin (g/dl)**								0.21			0.001
<2.5 (%)	‏-	‏-	‏-	‏-	0.0	100	0.0	‏-	0.0	100	‏-
2.5-3.5 (%)	‏-	‏-	‏-	‏-	27.1	68.8	4.2	‏-	12.7	87.3	‏-
>3.5 (%)	‏-	‏-	‏-	‏-	15.2	78.7	6.1	‏-	0.0	100	‏-

Low BMI (< 20kg/m^2^) was observed in 15.2% of females, and 22.7% of males. Furthermore, 8.7% of females and 3.5% males had high BMI (>30 kg/m^2^). There was a statistically significant relationship between BMI and sex (P =0.04). There was no significant association between gender and mortality (P=0.83) (Table 1).

Low serum albumin levels (2.5-3.5g/dl) were reported in 51% of patients with low BMI and 28.6% of patients with high BMI. While 71.4% of patients with high BMI and 49% of patients with low BMI had serum albumin levels higher than 3.5 g/dl. There was no significant relation between BMI and serum albumin levels (P=0.20) (Table 1). Of note, 16.7% of individuals with high BMI, 8.3% of individuals with low BMI and 4.9% of individuals with BMI 20-30 kg/m^2^ died during or shortly after surgery. There was no significant relation between these variables (P=0.20) (Table 1). Lower serum albumin levels (2.5-3.5g/dl) were reported in 40% of patients with more than 3 vessel grafts in CABG. Low BMI (<20 kg/m^2^) and high BMI (>30kg/m^2^) were identified in 14.3% and 11.1% of patients, respectively. However, there was no significant relation between variables.

Only one patient had low serum albumin levels (<2.5 mg/dl); so, we did not include him in later steps. Notably, this patient required reoperation due to bleeding after surgery and stayed in the ICU for more than 3 days. (Table 2). Of patients with normal serum albumin levels (>3.5 g/dl), only 2% required reoperation because of bleeding. However, of patients with low serum albumin levels (2.5-3.5g/dl) 12.9% needed reoperation. Considering P<0.001 and odds ratio=3.31, a significant positive correlation could be observed between decreased serum albumin levels and increased probability of reoperation due to bleeding (Table 2).

**Table 2:** Incidence percentage associated with different BMI and serum albumin in patients following CAB

	**Albumin (g/dl)**				**BMI (Kg/m** ^2^ **)**			
	**<2.5**	**2.5-3.5**	**>3.5**	***P***	**<20**	**20-30**	**>30**	***P***
**Re-exploration for bleeding**	100%	32%	12.9%	<0.001	22.7	16.7	21.1	0.502
**Deep sternal wound infection**	0.0	0.0	2.4	0.31	0.0	1.2	0.0	0.599
**Leg infection**	0.0	2.1	0.0	0.169	1.5	0.4	0.0	0.543
**CVA/TIA**	0.0	0.0	0.4	0.749	0.0	0.4	0.0	0.844
**MI**	0.0	2.1	0.0	0.169	0.0	0.4	5.3	0.023
**Low cardiac output**	0.0	2.1	0.6	0.543	1.5	1.6	0.0	0.858
**Pneumonia**	0.0	2.1	0.0	0.169	0.0	0.4	5.3	0.023
**Sepsis**	0.0	1	0.0	0.413	0.0	0.4	0.0	0.844
**ICU >3 days**	100	80.4	79.4	0.864	81.8	82.1	73.7	0.66
**Ventilatory support >1 day**	0.0	30.9	16.5	0.019	22.7	19.1	15.8	0.734
**Length of stay >10 days**	0.0	38.1	24.7	0.055	31.8	23.1	36.8	0.181
**Operative death**	0.0	12.7	0.0	<0.001	8.3	4.9	16.7	0.198

Of patients with low BMI (<20 kg/m^2^) and high BMI (> 30 kg/m^2^), 22.7% and 21.1% required reoperation because of bleeding, respectively. The relationship was not significant (P= 0.50) (Table 2).

Significant association did not exist between BMI and serum albumin levels in patients with CABG postoperative complications such as deep sternal region infection, leg infection, cerebrovascular accident/ transient ischemic attack, low cardiac output, sepsis, or requiring ICU more than 3 days (Table 2).

In addition, 5.3% of high BMI (>30 kg/m^2^) patients were affected by MI or pneumonia after operation while patients with low BMI (<20 kg/m^2^) did not develop these complications. There was a significant relationship between variables (P=0.02) (Table 2). Mechanical ventilation for more than 1 day was required in 30.9% of patients with low serum albumin levels (2.5-3.5g/dl); while 16.5% of patients with normal serum albumin levels (>3.5 g/dl) needed respiratory support for more than one day. There was a positive relation between serum albumin decrease from normal range and increased ventilator dependency period (P=0.019, odds ratio=2.27). Significant association was not found between BMI and need to ventilator for more than 1 day (Table 2). More than 10 days of hospitalization after the operation were required in 38.1% of patients with low serum albumin levels (2.5-3.5g/dl) (P=0.055). There was no relation between more than 10 days of hospitalization and BMI (Table 2).

12.7% of patients with low serum albumin levels (2.5-3.5g/dl) died during or after surgery. However, none of the patients with normal serum albumin levels (>3.5 g/dl) had such a condition. There was significant relation between variables. (Table 2).

Overall, 8.3% of patients with low BMI (<20 kg/m^2^) and 16.7% patients with high BMI (> 30 kg/m^2^) died during or after surgery. But the relationship between them was not significant (Table 2).

## Discussion

The youngest patient in our study with CABG was 30 years old. The mean age of the study population was 60.7 years; the ratio was lower than other studies conducted (for example, mean age of 70.4 years in a similar study). In this study, females were significantly more obese than men and males contributing to the majority of thin people. However, low serum albumin did not have association with gender. Moreover, mortality after CABG surgery was not affected by gender.

Old age had relation with decreased serum albumin and BMI. Majorities of the elderly patients were thin and had low serum albumin levels. However, age is not a risk factor for increased mortality after cardiac surgery. The simultaneous presences of other risk factors are needed for increased morbidity and mortality. Unlike the study of Kesek et al. (17), in our study obese patients (BMI> 30kg/m^2^) compared to patients with low BMI (<20 kg/m^2^) were at higher risk for MI after CABG which could increase mortality and adverse outcomes after the operation. This study showed that the risk of pneumonia after CABG increases in obese patients. However, patients with low BMI are less prone to this complication. The study did not find evidence of a direct effect of low or high BMI on postoperative complications including prolonged hospitalization or hospitalization in the intensive care unit for long time, sepsis, lower cardiac output, stroke, surgery site infection and reoperation due to bleeding.

In contrast with some of the previous studies^17^, in our study, patients with low serum albumin (2.5-3.5 g/dl) had higher mortality risk after cardiac surgery. These patients were also at higher risk of postoperative bleeding requiring reoperation after CABG. Low serum albumin levels are independent risk factor that increases the need for mechanical ventilation for more than 1 day after CABG despite correcting it before operation.

## Conclusion

Unlike other studies, low BMI and high BMI did not increase mortality after CABG in our study. Patients with a higher BMI are at increased risk of complications after heart surgery and their outcomes are worse. In patients with low serum albumin levels, mortality after CABG is high. These patients also have higher postoperative complications. Values of albumin levels are more prognostic for patient outcome and are of further importance.

## Ethical issues

The study was approved by the Ethics Committee of the University.

## Competing interests

Authors declare no conflict of interest in this study.
